# Books and Pamphlets Received

**Published:** 1886-03-20

**Authors:** 


### BOOKS AND PAMPHLETS RECEIVED.

*Coca Erythroxylen, and its Derivatives ;* a resume of their History, Botanical Origin, Production and Cultivation, Chemical Composition, Therapeutic Application, Physiological Action, and Medicinal Preparations ; embracing Reports on their employment in general and minor Surgery, Ophthalmology, Otology, Laryngology, Gynæcology, Genito-Urinary, Nasal, and Dental Surgery ; in the Treatment of the Alcohol and Opium Habits ; in general medicine, etc. Compiled by the Scientific Department of Parke, Davis & Co. Detroit and New York.

The above is an ably written work of 100 octavo pages. Every physician should fully acquaint himself with the new and wonderful preparation herein treated of, as it constitutes one of the most important additions to the armamentarium of the practitioner that has been made during the present century. The work may be obtained by addressing Parke, Davis & Co., Detroit, Mich.

*Magazine of American History.*

This is a monthly historical magazine. The copy before us is a large, beautifully gotten up magazine of 101 pages. It is designed to collate and present in neat, reliable form historical facts and information, not only of the late war but of American history in general. The present issue contains, among other interesting articles, the following : Champlain's American Experience in 1613;

Girty, the White Indian; The Trent Affair; Memorial Tribute to Chief Justice Read; Fortress Monroe; Ticonderoga. Terms of the magazine, $5 per annum. Address, "Magazine of American History," 30 La Fayette Place, New York.

*Climatology and Mineral Waters of the United States.* By A. N. Bell, A. M., M. D., editor of The Sanitarian; Member of the American Medical Association; of the American Public Health Association; of the American Climatological Association; of the Medical Society of the State of New York; formerly P. A. S. U. S. N., etc. New York: Wm. Wood. 1885.

Among the interesting topics treated in the above work we note: The Atmosphere, its extent and physical properties; Oxygen, and its properties; Ozone, etc.; Tests and Uses; Nitrogen, Ammonia, Carbonic Acid, and Electricity; Relation of Land and Water to Climate; Stability of Local Conditions of Climate; Heat; the Winds, Altitude, Atmospheric Pressure, Sea Coast places; Air, Forests; Climatological Topography in general; Climatological Topography and Mineral Springs of the Mississippi Basin; of the Western Highlands; of the Pacific Slope; the Weather; the Seasons; Relations of Climatology to Life Insurance, etc. The work is ably written and neatly gotten out, and contains 378 large octavo pages.

*Key to Smith's Diagram of Parliamentary Rules;* together with concise Hints and Directions for conducting the business of Deliberative Assemblies By Uriah Smith. Second edition, revised. Battle Creek, Mich.: Review and Herald Publishing Association. 1883.

This little book presents the subject in a most plain and condensed form possible. It contains an ingenious diagram of parliamentary rules, showing the relation of any motion to every other motion, and answering, at a glance, over five hundred questions in parliamentary practice. The work may well be examined and studied by every intelligent man who is liable to be called upon to preside at public meetings; and doctors would do well to procure one of these books, now that their good sense and patriotism is beginning to be appreciated by the public, as evinced in the large number of medical men who, of late years, are to be found in our State legislatures.

*Germ Action.* By Hiram Christopher, A M, M.D., Professor of Chemistry in the St. Joseph Medical College, St. Joseph, Mo.

A paper read before the North Western District Medical Society, St. Joseph, Mo.

*What Is Medicine?*

Annual address delivered before the American Academy of Medicine, at New York, October 28, 1885, by Albert L. Giron, A. M., M. D., Medical Director U. S. Navy, President of the Academy.

*Cocaine in Hay Fever.*

A lecture delivered at the Chicago Medical College, by Seth S. Bishop, M.D. Surgeon to the South Side Free Dispensary, and to the Illinois Charitable Eye and Ear Infirmary; Member of the American Medical Association.

